# A Bcl11b^N797K^ variant isolated from an immunodeficient patient inhibits early thymocyte development in mice

**DOI:** 10.3389/fimmu.2024.1363704

**Published:** 2024-03-01

**Authors:** Kazuaki Matsumoto, Kazuki Okuyama, Tom Sidwell, Motoi Yamashita, Takaho Endo, Naoko Satoh-Takayama, Hiroshi Ohno, Tomohiro Morio, Ellen V. Rothenberg, Ichiro Taniuchi

**Affiliations:** ^1^ Laboratory for Transcriptional Regulation, RIKEN Center for Integrative Medical Sciences (IMS), Yokohama Kanagawa, Japan; ^2^ Department of Pediatrics and Developmental Biology, Graduate School of Medical and Dental Sciences, Tokyo Medical and Dental University, Tokyo, Japan; ^3^ Division of Biology & Biological Engineering, California Institute of Technology, Pasadena, CA, United States; ^4^ Genome Platform, RIKEN Center for Integrative Medical Sciences (IMS), Yokohama Kanagawa, Japan; ^5^ Laboratory for Intestinal Ecosystem, RIKEN Center for Integrative Medical Sciences (IMS), Yokohama Kanagawa, Japan

**Keywords:** IEIS, immune deficiency, BCL11B, mis-sense variant, T cell

## Abstract

BCL11B is a transcription factor with six C_2_H_2_-type zinc-finger domains. Studies in mice have shown that Bcl11b plays essential roles in T cell development. Several germline heterozygous BCL11B variants have been identified in human patients with inborn errors of immunity (IEI) patients. Among these, two *de novo* mis-sense variants cause asparagine (N) to lysine (K) replacement in distinct zinc-finger domains, BCL11B^N441K^ and BCL11B^N807K^. To elucidate the pathogenesis of the BCL11B^N807K^ variant, we generated a mouse model of BCL11B^N807K^ by inserting the corresponding mutation, *Bcl11b^N797K^*, into the mouse genome. In *Bcl11b^+/N797K^* mice, the proportion of immature CD4^−^CD8^+^ single-positive thymocytes was increased, and the development of invariant natural killer cells was severely inhibited in a T-cell-intrinsic manner. Under competitive conditions, γδT cell development was outcompeted by control cells. *Bcl11b^N797K/N797K^* mice died within one day of birth. Recipient mice reconstituted with *Bcl11b^N797K/N797K^* fetal liver cells nearly lacked CD4^+^CD8^+^ double-positive thymocytes, which was consistent with the lack of their emergence in culture from *Bcl11b^N797K/N797K^* fetal liver progenitors. Interestingly, *Bcl11b^N797K/N797K^* progenitors gave rise to aberrant c-Kit^+^ and CD44^+^ cells both *in vivo* and *in vitro*. The increase in the proportion of immature CD8 single-positive thymocytes in the Bcl11b^N797K^ mutants is caused, in part, by the inefficient activation of the *Cd4* gene due to the attenuated function of the two *Cd4* enhancers via distinct mechanisms. Therefore, we conclude that immunodeficient patient-derived Bcl11b^N797K^ mutant mice elucidated a novel role for Bcl11b in driving the appropriate transition of CD4^−^CD8^−^ into CD4^+^CD8^+^ thymocytes.

## Introduction

Inborn errors of immunity (IEI) are characterized by defects in the function of the immune system. Genetic defects, including mis-sense variants, are often involved in the pathogenesis of IEI (1). Among the genes identified as causal factors for IEIs, variants encoding factors governing primary lymphocyte development, such as *IL2RG* and *RAG1/2*, are often associated with the severe combined immune-deficient (SCID) phenotype in human patients. Transcription factors (TFs) play essential roles in decoding the lymphocyte developmental program embedded in the genome; their dysfunction also influences lymphocyte development. Recent studies have isolated gene variants in the IKAROS family zinc finger (IKZF) genes and STAT5 from patients with B or T lymphocyte deficiency.

Bcl11b is a zinc-finger (ZnF) type transcription factor. There are one atypical CCHC-type and six C_2_H_2_-type ZnF motifs in Bcl11b. Among the six C_2_H_2_ ZnFs, the first three are located in the middle of Bcl11b, and the last three are located at the C-terminal ends (2). Studies in mice have shown that Bcl11b is essential in controlling T lymphocyte development (3–5). Primary T lymphocyte development occurs in the thymus after early T cell progenitors (ETP), which are generated extrathymically, migrate to the thymus during embryogenesis. The developmental stages of thymocytes are classically divided into four populations according to CD4 and CD8 coreceptor expressions. The most immature thymocytes do not express neither CD4 nor CD8, and are thereby referred to as CD4^−^CD8^−^ double-negative (DN) thymocytes. DN thymocytes are further divided into CD44^+^CD25^−^ DN1, CD44^+^CD25^+^ DN2, CD44^−^CD25^+^ DN3, and CD44^−^CD25^−^ DN4 thymocytes. *Bcl11b* gene expression is induced at the CD44^+^CD25^+^ DN2 stage, where the Bcl11b protein plays a crucial role in commitment toward the T-lineage via suppressing developmental potency to non-T lineage cells, such as myeloid cells (5–7). Bcl11b is also indispensable for the T cell receptor (TCR) signal-mediated selection of CD4^+^CD8^+^ double-positive (DP) thymocytes, a process known as positive selection (8). Following positive selection, major histocompatibility complex class I (MHC-I) and MHC-II restricted thymocytes are differentiated into CD4^−^CD8^+^ and CD4^+^CD8^−^ single positive (SP) thymocytes, which are committed to the cytotoxic and helper lineages, respectively. Bcl11b is important for the helper/cytotoxic lineage choice by regulating the lineage-specifying transcription factors, *Thpok* (also known as *Zbtb7b*) and *Runx3* (9). Additionally, Bcl11b is essential for the development of regulatory T cells (10, 11) and invariant natural killer T (iNKT) cells in the thymus (12). Studies in mice have revealed that Bcl11b regulates the development of different types of T cells. Furthermore, Bcl11b is required for the development of group 2 innate lymphoid cells (ILC2s) in mice by regulating ILC2 progenitor development in fetuses (13, 14).

The importance of Bcl11b in T cell and ILC2 development in humans has been confirmed by isolating several genetic variations of the *BCL11B* gene from human patients (15, 16). Among human patients with immunodeficiency 49 (IMD49) and intellectual developmental disorder with speech delay, dysmorphic facies, and T cell abnormalities (IDDSFTA), several *BCL11B* variants (five missense, two non-sense, and 11 frameshift variants) have been identified (15–23). Among those, the *de novo* variant that results in the replacement of asparagine (N) with lysine (K) within the second ZnF motif, referred to as BCL11B^N441K^, was the first *BCL11B* variant proposed to cause T-lymphocyte deficiency (15). Interestingly, another variant causing the replacement of the N807 residue in the fourth ZnF with K, referred to as BCL11B^N807K^, was identified in one patient (16). Both patients showed low T cell receptor excision circles (TRECs) at birth even though they were only heterozygous for variants *BCL11B^N441K^* or *BCL11B^N807K^*. Thus, the N-to-K replacement in these distinct ZnF domains of BCL11B will likely impair its function in a dominant-negative way. However, whether impaired T cell development by these similar but distinct BCL11B variants, BCL11B^N441K^ or BCL11B^N807K^, is caused by a similar or distinct pathogenesis remains elusive.

In this study, we generated and examined a mouse model harboring the corresponding mutation in the murine Bcl11b protein, *Bcl11b^N797K^*, to elucidate the pathogenesis of the BCL11B^N807K^ variant. We found that early T cell development in the thymus was impaired at the DN2 to DN3 and DN to DP transitional stages in *Bcl11b^+/N797K^* mice. Thus, our *Bcl11b^N797K^* mouse model is a good model for elucidating the pathogenesis of IEIs caused by the BCL11B^N807K^ variant.

## Materials and methods

### Mice

A DNA fragment harboring C to A nucleotide change that generates Bcl11b^N797K^ protein was generated by overlap PCR, and replaced with *wt* exon 4 in the target vector, which was used to generate the *Bcl11b^flo^* ^x^ allele (9). The targeting vector for Bcl11b^N797K^ mutation was transfected into M1 embryonic stem (ES) cells as previously described (9), and ES clones which underwent homologous recombination were identified by PCR with an appropriate primer set. *Bcl11b^+/N797K^* mice were generated by crossing C57BL/6NJcl mice purchased from CLEA Japan (Shizuoka, Japan) with chimeric mice generated from ES clones with *Bcl11b^+/N797K^* genotype. The *Bcl11b<σπ>Δ</σπ>* allele was generated from the *Bcl11b^fl^ allele* (9) by crossing *Bcl11b^+/fl^* mice with *EIIa-Cre* transgenic mice that were purchased from Jackson laboratory (#003314). *Cd4^ΔS^ mice* was previously described (24). CD45.1 congenic mice and *Rag1*-deficient mice were obtained from Jackson Laboratories. All mice were maintained in the animal facility at the RIKEN IMS. All animal procedures were in accordance with institutional guidelines for animal care and the protocol (2020–026) approved by the Institutional Animal Care and Use Committee of RIKEN Yokohama Branch. All the mice were sacrificed by euthanasia with CO_2_ overdose following anesthesia using isoflurane.

### Flow cytometry

Thymus, spleen, lymph nodes and other organs were harvested from mice at various, mashed and passed through 100 μm pore cell strainer to make single cell suspensions. After hemolysis with ACK Lysing Buffer (Thermo Fisher Scientific, MA), cells were washed with ice-cold staining buffer (D-PBS (-), 2mM EDTA, 0.05% NaN_3_, and 2% FBS) followed by blocking with 2.5 μg/ml of 2.4G2 antibody (BD Biosciences, NJ). The small intestine was dissected, and intestinal contents were washed out with cold RPMI-1640 medium (SIGMA). The intestine was cut into approximately 1.0 cm pieces and incubated in the RPMI-1640 medium containing 5 mM EDTA and 2% fetal bovine serum (FBS) for 15 min at 37°C, followed by another cycle of incubation in RPMI-1640 medium with 2% FBS. Remaining intestinal tissues were cut into small pieces and digested with 1.0 mg/ml collagenase (SIGMA) suspended in RPMI-1640 medium for 15 min at 37°C. The resultant supernatants from the collagenase digestion were collected and passed through a 100 μm cell strainer after 3 cycles of these steps. The cells were subjected to Percoll (G.E. Healthcare) gradient separation and lymphocytes in the interphase were collected and proceeded for the flow cytometric analysis.

Surface molecules were stained with specific antibodies by incubating for 30 min on ice. The following antibodies for surface molecules were purchased from BD Biosciences or Thermo Fisher Scientific; B220 (clone: RA3-6B2), CD3ε (clone: 145-2C11), CD4 (clone: RM4-5), CD8α (clone: 53-6.7), CD11b (clone: M1/70), CD19 (clone: 1D3), CD24 (clone: M1/69), CD25 (clone: PC61.5), CD44 (clone: IM7),CD45 (clone: 30-F11, or clone:104), CD45.1 (clone: A20), CD45.2 (clone: 104), CD62L (clone: MEL-14), CD127 (clone:A7R34), c-Kit (clone: 2B8), Gr-1 (clone: RB6-8C5), IL-7Rα (clone: A7R34), ST2 (clone: DIH4), Sca1 (clone:E13-161.7), NK1.1 (clone: PK136), TCRβ (clone: H57-597) and γδTCR (clone: GL3). α-galactosyl ceramide (a-GalCer) loaded CD1d tetramer was prepared by mixing recombinant dimeric mouse CD1d:Ig fusion protein (BD Biosciences, DimerX I). In order to investigate intracellular molecules, cells were fixed and permeabilized by use of Transcription Factor Buffer Set (BD Biosciences) following surface staining. Transcription factors were detected with following antibodies purchased from BD Biosciences or abcam (Cambridge, UK); Bcl11b (clone: 25B6), Foxp3 (clone: FJK-16s), Gata3 lone: 16E10A23), Rorγt (clone: Q31-378), and Thpok (clone: T43-94). Dead cells were distinguished by use of 7-amino-actinomycin D (7-AAD) (BD Biosciences) and LIVE/DEAD^®^ Fixable Dead Cell Stain (Thermo Fisher Scientific) for general and intracellular staining, respectively. Multi-color flow cytometric analysis was performed using BD FACSCanto II™ (BD Biosciences), and data was processed with FlowJo™ software (BD Biosciences). Cell subsets were sorted using a BD FACSAria™ III (BD Biosciences).

### Competitive mixed bone marrow chimera, and fetal liver transplantation

Ten million bone marrow cells were isolated from femur of mice at 6 weeks of age with either CD45.2 *Bcl11b^+/+^* or *Bcl11b^+/N797K^* genotype by flushing, and mixed with equal number of CD45.1 *Bcl11b^+/+^* bone marrow cells in 200 μl of D-PBS (-). Bone marrow cells were intravenously injected into lethally irradiated (9.5 Gy) *Rag1*-deficient recipients. Fetal liver (FL) cells isolated from E13.5 or 14.5 *Bcl11b^+/+^* and *Bcl11b^N797K/N797K^* embryos were mashed and passed through 100 μm strainer to prepare single cell suspension. FL cells were washed with ice-cold D-PBS (-) once, suspended in 200 μl of D-PBS (-), and injected into lethally irradiated (9.5 Gy) *Rag1*-deficient mice intravenously. Drinking water containing antibiotics (1 μg/ml neomycin and 100 unit/ml polymyxin B) was supplied to recipient mice for the first two weeks post-transplantation. Three months after the transplantation, lymphoid organs were isolated from mice and flow-cytometric analyses were performed.

### OP9 culture and cell counting in OP9 culture

Frozen fetal liver (FL) samples were thawed and cultured in OP9 media (alpha MEM [Gibco] supplemented with 20% FBS, 50μM β-ME (Sigma), penicillin-streptomycin-glutamine supplement [Gibco]) overnight with 10 ng/ml IL-7 and Flt3L and 5 ng/ml KitL (Peprotech). The following day, cells were depleted by biotinylated antibodies (Biolegend) and anti-biotin magnetic beads (Miltenyi) for lineage markers (TCRβ, CD19, CD11c, NK1.1, Ter119, γδTCR, Gr-1). Remaining Lineage-negative cells were subsequently cultured on an OP9-DLL1 cell monolayer in OP9 medium with IL-7 and Flt3L (10ng/ml until day 5, 5 ng/ml until day 8, 1 ng/ml subsequently) and split every 2-3 days.

On harvesting, a known fraction of the culture was immediately removed, and mixed with a known volume of a resuspended mix of PBS (Gibco), SytoxBlue (Invitrogen) and single colour Calibrite compensation beads (BD). The number of beads in the counting mix was established with a hemocytometer. Bead/Cell mixes were vigorously resuspended by pipetting prior to acquisition by flow cytometry (MACSQuantify, Miltenyi). On flow cytometric analysis (FlowJo software, BD) the ratio of viable (SytoxBlue-negative) and non-OP9 (GFP-negative) cells to beads was established, and the absolute number of cells calculated. The dilution factor of each split was accounted for to calculate absolute cell numbers, and these were normalized to day 5 (as 100%) to account for cell number differences between samples. Population frequencies of total viable cells were extracted by flow cytometry and multiplied by these normalized absolute cell counts.

### Culture of DN2 cells on TSt-4/Dll4 stroma cells

Total thymocytes were harvested from lethally irradiated *Rag1*-deficient recipients, in which thymocyte development was reconstituted with *Bcl11b^+/+^* and *Bcl11b^N797K/N797K^* FL cells at eight weeks after the transplantation, and CD4^−^CD8α^−^CD44^+^CD25^+^ DN2 thymocytes were purified by cell sorting. Cells were seeded on TSt-4/Dll4 feeder cells, and cultivated in RPMI1640 medium supplemented with 10% FBS, 50 μM β-ME, antibiotics, and cytokine cocktail (5 ng/ml each of KitL, Flt3L, and IL-7). Five days after, cells were harvested by vigorous pipetting and were passaged to new feeder cells. Flow cytometric analyses were performed on day 5^th^ and 9^th^. Dead cells and feeder cells were distinguished from hematopoietic cells by 7-AAD staining and CD45 expression. The absolute numbers of hematopoietic cells in culture were calculated, and were normalized to day 0 (as 100%).

### Assay for transposase-accessible chromatin sequencing

One hundred thousand target cells (DN [CD24^+^TCRβ^−^CD4^−^CD8α^−^], ISP [CD24^+^TCRβ^−^CD4^−^CD8α^+^] and DP [CD24^+^TCRβ^−^CD4^+^CD8α^+^] thymocytes) were purified by cell sorting. Cells were washed with ice-cold D-PBS(-), and ATAC libraries were prepared by use of ATAC-Seq Kit (Active Motif, CA) according to manufacturer’s protocol. To deplete large DNA fragments, prepared library DNA solution was mixed with x 0.65 volume of SPRIselect beads (Beckman Coulter, CA) and supernatant was collected. Supernatant was then mixed with x 0.65 volume of beads, and DNA fragments captured on the beads were eluted with 0.1x TE buffer resulted in an enrichment of fragments with 150-500 bp length. Fragment size was validated by Agilent, 2100 Bioanalyzer and High Sensitivity DNA Kit (Agilent, CA). Libraries were quantified by quantitative PCR using KAPA Library Quantification Kit (Roche). Sequencing was performed with Illumina HiSeqX (Illumina, CA) at Macrogen (Seoul, South Korea).

### Data and statistical analyses

ATAC-sequence reads were trimmed and their quality were checked using trimgalore (version 0.6.7, https://github.com/FelixKrueger/TrimGalore) and then mapped to the mm10 genome using bowtie2 (version 2.4.5, https://bowtie-bio.sourceforge.net/bowtie2/index.shtml). Mapped reads were normalized by total reads and window size and were expressed as RPKM (reads per kilobase per million reads). Statistical analysis was performed by F-test and unpaired Students’ *t* test with or without Welch’s correction using GraphPad Prism 7 (Graphpad software, CA) or Excel (Microsoft, WA). ATAC-Seq data are available at under accession number SRP473952.

## Results

### Accumulation of immature CD8 SP thymocytes in Bcl11b^+/N797K^ mice

The position of the N807 residue replaced with K in human patients harboring the BCL11B^N807K^ variant corresponded to the N797 position in the mouse Bcl11b protein (**Supplementary Figure 1A**). Therefore, we introduced a C-to-A single nucleotide mutation via gene targeting using mouse ES cells in the mouse *Bcl11b* gene, hereafter referred to as the *Bcl11b^N797K^* allele, which produces the Bcl11b^N797K^ protein (**Supplementary Figure 1B**). A mouse strain harboring the *Bcl11b^N797K^* mutation was established through germline transmission from chimeric mice generated from a *Bcl11b^+/N797K^* ES cell clone (**Supplementary Figure 1C**). We first noticed that heterozygous *Bcl11b^+/N797K^* founders showed slight growth retardation and did not gain normal weight at weaning (**Supplementary Figure 1D**). *Bcl11b^+/N797K^* mice also tended to die at one week of age, and only half of then survived at six weeks of age on a normal diet (**Supplementary Figure 1E**). Our RNA-seq data using thymocytes from *Bcl11b^+/N797K^* mouse confirmed that *Bcl11b* mRNA was transcribed equally from both the *Bcl11b^+^* and *Bcl11b^N797K^* alleles (**Supplementary Figure 1F**), and Bcl11b protein expression in DP thymocytes was comparable between *Bcl11b^+/+^* and *Bcl11b^+/N797K^* mice (**Supplementary Figure 1G**).

**Figure 1:**
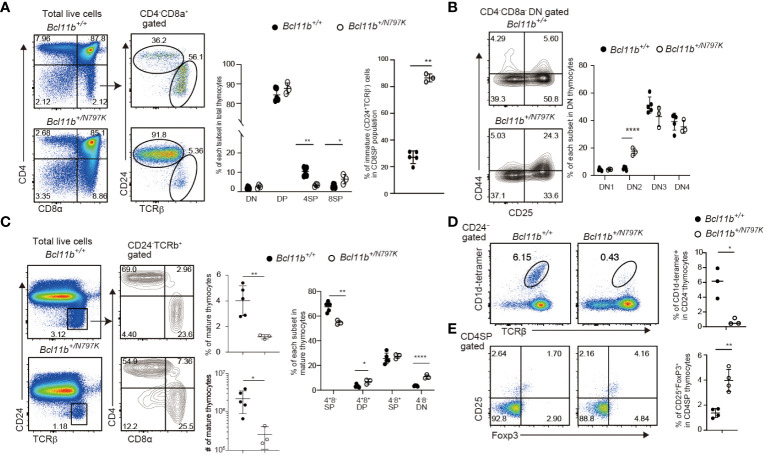
Impaired thymocyte development in *Bcl11b^+/N797K^* mice. **(A)**. Representative pseudo-color plots showing CD4 and CD8α expressions in total thymocytes and CD24 and TCRβ expression in CD4^−^CD8α^+^ thymocytes obtained from *Bcl11b^+/+^* and *Bcl11b^+/N797K^* mice. Graphs show the frequency of each thymocyte subset indicated. **(B)**. Representative contour plots showing CD25 and CD44 expressions in CD4^−^CD8α^−^ DN thymocytes. Graph shows the frequency of each DN thymocyte subset indicated. **(C)**. Representative pseudo-color plots of CD24 and TCRβ expression in total thymocytes. Right contour plots show CD4 and CD8α expressions in CD24^−^TCRβ^+^ mature thymocytes from *Bcl11b^+/+^* and *Bcl11b^+/N797K^* mice. Graph shows the frequency and the number of mature thymocyte and the frequency of each subset indicated in mature thymocytes. **(D)**. The upper pseudo-color plots showing staining pattern with TCRβ and CD1d tetramer loaded with αGalCer to detect iNKT cells in CD24^−^ thymocytes. Graph shows the frequency of iNKT cells in CD24^−^ thymocytes. The lower pseudo-color plots showing CD25 and Foxp3 expression in CD4^+^CD8α^−^ thymocytes and graph shows the frequency of CD25^+^Foxp3^+^ Treg cells in CD4^+^CD8α^−^ thymocytes. All graphs show mean value ± SD. Unpaired Students’ *t*-tests were performed. *: *p* < 0.05, **: *p* < 0.005, ****: *p* < 0.00005.

Since the patient with *de novo* heterozygosity for the *BCL11B^N807K^* variant showed impaired T cell development (16), we first examined T cell development in *Bcl11b^+/N797K^* mice at four weeks of age using flow cytometry. Thymocytes cellularity were reduced to approximately one-tenth in *Bcl11b^+/N797K^* mice compared to *Bcl11b^+/+^* mice (**Supplementary Figure 2A**). The CD4 and CD8α expression profiles showed an increase in the frequency of the CD4^−^CD8^+^ SP population and a decrease in the frequency of the CD4^+^CD8^−^ SP population (**Figure 1A**). The CD4^−^CD8^+^ SP population consists of immature (CD24^+^TCRβ^lo^) and mature (CD24^−^TCRβ^hi^) thymocytes. Analyses of CD24 and TCRβ expression revealed that the frequency of the immature CD24^+^TCRβ^lo^ CD8 SP thymocytes, hereafter referred to as ISP8 thymocytes, was elevated to more than 80% in the thymus of *Bcl11b^+/N797K^* mice (**Figure 1A**). Accumulation of ISP8 thymocytes by the *Bcl11b^N797K^* protein indicates that thymocytes differentiation was impaired by the Bcl11b^N797K^ mutant at the transition from the CD4^−^CD8^−^ DN to the CD4^+^CD8^+^ DP stage. In the CD4^−^CD8^−^ DN thymocytes population, the frequency of the CD44^+^CD25^+^ DN2 subset was increased with a tendency for a decreased percentage of the CD44^−^CD25^+^ DN3 subset in *Bcl11b^+/N797K^* mice compared to *Bcl11b^+/+^* mice (**Figure 1B**). We also noticed that the percentage of mature (CD24^−^TCRβ^hi^) thymocytes was lower in *Bcl11b^+/N797K^* mice than in *Bcl11b^+/+^* mice (**Figure 1C**). In addition, the percentages of CD4^−^CD8α^−^ and CD4^+^CD8α^+^ cells in the mature thymocyte population were elevated in *Bcl11b^+/N797K^* mice, as was observed in the mutant mice lacking the last ZnF motif in the Bcl11b protein (9).

Bcl11b is also essential for the development of iNKT (12) and Foxp3^+^ regulatory T (Treg) cells (10), two lineages that are known to be positively agonist-selected (25). The frequency of iNKT cells that were detected as TCRβ^+^ cells bound by αGalCer-loaded CD1d-dimer in the CD24^−^ mature thymocyte population was significantly lower in the thymus of *Bcl11b^+/N797K^* mice than in *Bcl11b^+/+^* mice (**Figure 1D**). In the thymic iNKT population, there was a decrease in the frequency of NK1.1^−^CD44^+^ stage 2 subset in the *Bcl11b^+/N797K^* mice compared with controls (**Supplementary Figure 2B**). Thus, Bcl11b^N797K^ mutant interferes with iNKT differentiation at the early developmental stage. In contrast, the frequency of Foxp3^+^ Treg cells in the CD4 SP thymocyte population was higher in *Bcl11b^+/N797K^* mice than in *Bcl11b^+/+^* mice (**Figure 1E**). These results indicate that the mutant Bcl11b^N797K^ protein perturbs early T cell development, as expected in a human patient (16). Bcl11b is also required for the development of ILC2s in mice (13, 14), and some human patients harboring other BCL11B variants showed a reduction in ILC2s in their blood (16, 18). We therefore next examined ILC2 development in our mutant mice. The frequency of Gata3^+^Rorγt^-^ ILC2s among the small intestine CD45^+^CD3ε^−^IL-7Rα^+^ lamina propria cells was modestly, though statistically, lower in *Bcl11b^+/N797K^* than in *Bcl11b^+/+^* mice (**Supplementary Figure 2C**). Thus, as observed in the reduction of ILC2s in the blood of *BCL11B^+/N807K^* human patients, the corresponding mis-sense mutation *Bcl11b^N797K^* in mice likely impairs ILC2 development.

In the peripheral lymphoid tissues, the total number of splenocytes was less than one-fifth in *Bcl11b^+/N797K^* mice than in *Bcl11b^+/+^* mice (**Supplementary Figure 2D**), presumably due to their smaller body sizes. As expected, the frequency of TCRβ^+^ T cells in the spleen was decreased in *Bcl11b^+/N797K^* mice (**Supplementary Figure 2D**). Although, the CD4/CD8 ratio was comparable between *Bcl11b^+/N797K^* and *Bcl11b^+/+^* mice, both CD4^+^ and CD8^+^ splenic T cells tended to differentiate into CD44^+^ memory-phenotype T cells (**Supplementary Figure 2E**). For instance, the frequency of the CD44^+^CD62L^+^CD8^+^ T cell subset (CD8^+^ T central memory; TCM) and CD44^+^CD62L^−^ CD4^+^ T subset (CD4^+^ T effector memory; TEM) and CD44^+^CD62L^−^ CD8^+^ T cell subset (CD8^+^ TEM) were elevated, while that of the CD44^−^CD62L^+^ naïve T cell subset was reduced in *Bcl11b^+/N797K^* mice (**Supplementary Figure 2E**). This increase in memory phenotype T cells has been reported as a BCL11B-related disorder (BCL11B-RD) obseved in human patients (26).

### Bcl11b^N797K^ perturbs T cell development in a T-cell intrinsic manner

**Figure 2:**
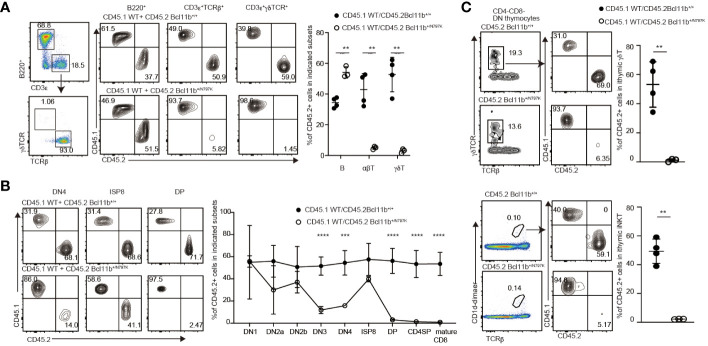
T cell intrinsic effects by Bcl11b^N797K^ variant for impaired T cell development. **(A)** Left pseudo-color plot showing a gating strategy and right contour plots showing CD45.1 and CD45.2 expressions in splenic B cells, αβT cells and γδT cells from recipient mice transplanted CD45.1^+^ *Bcl11b^+/+^* bone marrow cells with either CD45.2^+^ *Bcl11b^+/+^* or *Bcl11b^+/N797K^* bone marrow cells. Graph presents the summary of the frequency of CD45.2^+^ cells in the indicated cells. **(B)** Representative contour plots showing CD45.1 and CD45.2 expressions in selected thymocyte subsets. Graph presents mean ± S.D. and shows the summary of the percentage of CD45.2^+^ cells in each thymocyte subsets from recipients transplanted either CD45.2^+^ *Bcl11b^+/+^* (n=4) or *Bcl11b^+/N797K^* (n=3) bone marrow cells with CD45.1^+^ *Bcl11b^+/+^* bone marrow cells. **(C)** Representative contour plots showing CD45.1 and CD45.2 expressions in thymic γδT cells (upper) and iNKT cells (lower) from recipient mice. Gating strategy is shown at the left. Graph shows the summary of the frequency of CD45.2^+^ cells in γδT cells and iNKT cells. All the graphs show mean value ± SD. Unpaired Students’ *t*-tests were performed. **: *p* < 0.005, ***: *p* < 0.0005.

To examine whether T cell development in *Bcl11b^+/N797K^* mice is impaired in a hematopoietic cell-intrinsic manner and the developmental potency of *Bcl11b^+/N797K^* progenitors in a competitive setting against *Bcl11b^+/+^* cells, we performed mixed bone marrow chimera experiments. A mixture of CD45.1^+^ *Bcl11b^+/+^* bone marrow cells and either CD45.2^+^ *Bcl11b^+/+^* or *Bcl11b^+/N797K^* bone marrow cells was injected into lethally irradiated *Rag1*-deficient mice (**Supplementary Figure 3A**). Eight weeks after transplantation, the ratio of CD45.2^+^ to CD45.1^+^ cells was examined among several types of hematopoietic cells. Compared with the Gr-1^+^ myeloid and B220^+^ B lymphocyte populations in the circulating blood, the frequency of CD45.2^+^ cells in CD3ε^+^ T cells was lower in recipient mice that received CD45.2^+^ *Bcl11b^+/N797K^* bone marrow cells (**Supplementary Figure 3B**). In the spleen, we confirmed that *Bcl11b^+/N797K^* progenitors gave rise to B220^+^ B lymphocytes, whereas the frequency of CD45.2^+^ cells in both CD3ε^+^TCRβ^+^ and CD3ε^+^γδTCR^+^ cells was significantly reduced in recipients that received CD45.2^+^ *Bcl11b^+/N797K^* bone marrow cells (**Figure 2A**). This reduction was more severe in the CD3ε^+^γδTCR^+^ population than in the CD3ε^+^TCRβ^+^ population. Thus, the developmental potency to generate mature T cells in *Bcl11b^+/N797K^* progenitors was nearly outcompeted by the control progenitors under equally mixed settings. Next, we examined at which T developmental stages, *Bcl11b^+/N797K^* progenitors were outcompeted. In the thymus, while the ratio of CD45.2^+^ *Bcl11b^+/N797K^* cells to CD45.1^+^ *Bcl11b^+/+^* cells was nearly one in the DN1 population, it started to decline from the CD44^+^CD25^+^ DN2a stage and was significantly lower at the CD44^−^CD25^+^ DN3 stage (**Figure 2B**). These results indicated that the Bcl11b^N779K^ mutant protein may impair the generation of DN2a thymocytes. Interestingly, the frequency of CD45.2^+^ cells increased at the ISP8 stage, followed by a sharp decrease at the DP stage (**Figure 2B**). The accumulation of CD45.2^+^ *Bcl11b^+/N797K^* ISP8 thymocytes relative to the control CD45.1^+^ *Bcl11b^+/+^* cells, which stems from a developmental block at the transition from the DN4 to DP stage by the Bcl11b^N797K^ protein, is likely a cause of the increase in the CD45.2^/^CD45.1 ratio at the ISP8 stage. Thus, the potency of generating DP thymocytes from *Bcl11b^+/N797K^* progenitors was outcompeted at this transitional stage.

We also examined the ratio of CD45.2^+^ to CD45.1^+^ cells in thymic γδT and iNKT cell populations and found that *Bcl11b^+/N797K^* progenitors were nearly outcompeted by control progenitors in generating these cells (**Figure 2C**). Previous studies have shown that Bcl11b plays an important role in presenting glycolipid antigens on CD1d during iNKT cell development (12). However, the distinct scarcity of mutant DP cells in our model rules out a role for impaired antigen presentation to iNKT-destined cells. As such, our results confirmed that a cell-autonomous mechanism was a major cause of impaired iNKT development in the *Bcl11b^+/N797K^* mice.

### Loss of DP thymocytes in Bcl11b^N797K/N797K^ neonatal mice

**Figure 3:**
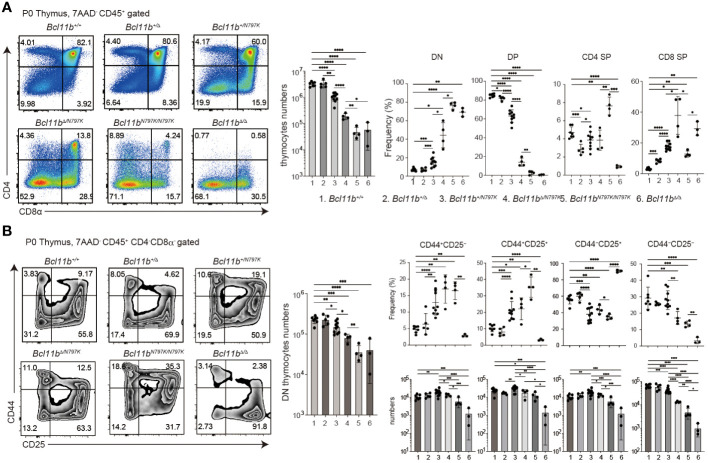
Impaired thymocytes differentiation in *Bcl11b^N197K/N797K^* new born mice. **(A)**. Representative pseudo-color plots showing CD4 and CD8α expressions in total thymocytes of new born mice with indicated genotypes. Numbers of total thymocytes and the frequency of CD4^−^CD8α^−^ (DN), CD4^+^CD8α^+^ (DP), CD4^+^CD8α^−^ (CD4 SP) and CD4^−^CD8α^+^ (CD8 SP) thymocyte subset are shown in the graphs. **(B)** Representative pseudo-color plots showing CD25 and CD44 expressions in live CD45^+^CD4^−^CD8α^−^ thymocytes of new born mice with indicated genotypes. Numbers of CD45^+^CD4^−^CD8α^−^ thymocytes and the frequency and cell numbers of CD44^+^CD25^−^, CD44^+^CD25^+^, CD44^−^CD25^+^ and CD44^−^CD25^−^ thymocyte subset are shown. All the graphs show mean value ± SD and 1, 2, 3, 4, 5, and 6 in the graph are *Bcl11b^+/+^*, *Bcl11b^+/Δ^*, *Bcl11b^+/N797K^*, *Bcl11b^Δ/N797K^*, *Bcl11b^N797K/N797K^* and *Bcl11b^Δ/Δ^*, respectively. Unpaired *t*-tests were performed. *: *p* < 0.05, **: *p* < 0.005, ***: *p* < 0.0005, ****: *p* < 0.00005.

We analyzed offspring obtained by intercrossing *Bcl11b^+/N797K^* heterozygous mice and found that there were no live *Bcl11b^N797K/N797K^* homozygous neonates at one week of age (P7), although *Bcl11b^N797K/N797K^* homozygous mice were born alive at birth (P0) (**Supplementary Figure 3C**). This indicates that *Bcl11b^N797K/N797^* mice died during the neonatal period, as was observed in *Bcl11b*-defcient *Bcl11b^Δ/Δ^* mice (27). Therefore, we examined thymocyte development in P0 neonates including *Bcl11b^+/N797K^* obtained by crossing *Bcl11b^+/N797K^* and *Bcl11b^+/Δ^* mice, as well as *Bcl11b^Δ/Δ^* mice. Compared to *Bcl11b^+/+^* and *Bcl11b^+/Δ^* neonates, the total number of thymocytes was lower in *Bcl11b^+/N797K^* neonates, and was further reduced in *Bcl11b^Δ/N797K^* neonates (**Figure 3A**), in which only the mutant Bcl11b^N797K^ protein was expressed. Thymocytes cellularity was the lowest in *Bcl11b^N797K/N797K^* and *Bcl11b^Δ/Δ^* neonates and was comparable between these two genotypes (**Figure 3A**). The CD4 and CD8α expression profiles revealed a clear increase in the frequency of CD4^−^CD8^−^ DN thymocytes in the thymus of neonates harboring the *Bcl11^N797K^* allele (**Figure 3A**). The generation of CD4^+^CD8^+^ DP thymocytes was significantly inhibited in *Bcl11b^Δ/N797K^* neonates. A small CD4^+^CD8^+^ DP thymocyte subset was emerged in *Bcl11b^N797K/N797K^* neonates, whereas these DP thymocytes were absent in *Bcl11b^Δ/Δ^* neonates (**Figure 3A**). In line with the decrease in total thymocyte number, the number of CD4^−^CD8^−^ DN thymocytes was also decreased by the presence of the *Bcl11b^N797K^* protein (**Figure 3B**). Moreover, CD44 and CD25 expression profile of the CD4^−^CD8^−^ DN population revealed that the frequency of CD44^+^CD25^−^ DN1 and CD44^+^CD25^+^ DN2 subsets was increased in *Bcl11b^+/N797K^*, *Bcl11b^Δ/N797K^* and *Bcl11b^N797K/N797K^* neonates (**Figure 3B**). However, the cell number of these subsets was lower in all these mice than in wild-type mice. Thus, early thymocyte development in *Bcl11b^N797K/N797K^* new-born mice was similar, but not identical, to that in *Bcl11b^Δ/Δ^* mice, suggesting that Bcl11b^N797K^ variant protein retains its Bcl11b function to some extent.

### Bcl11b^N797K/N797K^ progenitors retain potency to give rise to T cells

**Figure 4:**
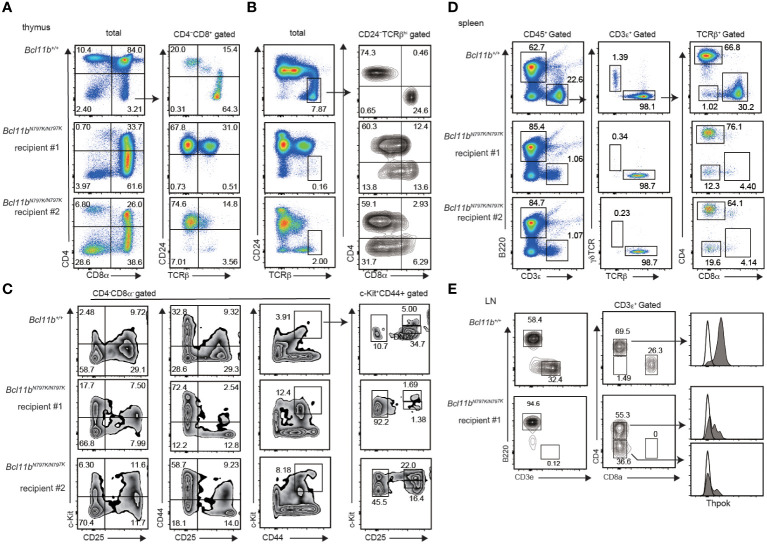
Impaired T cell development from *Bcl11b^N797K/N797K^* progenitors in Rag1-deficient recipients. **(A–C)**. Flowcytometry of thymocytes of Rag1-deficient recipient injected with *Bcl11b^+/+^* and *Bcl11^N797K//N797K^* fetal liver cells. pseudo-color plots showing CD4 and CD8α expression in total thymocytes and CD24 and TCRβ expression in CD4^−^CD8α^+^ thymocytes **(A)**. pseudo-color plots showing CD24 and TCRβ expression in total thymocytes and contour plots showing CD4 and CD8α expression in CD24^−^TCRβ^+^ mature thymocytes **(B)**. Contour plots showing CD44, CD25 and c-Kit expression in CD4^−^CD8α^−^ thymocytes. Right plots showing CD25 and c-Kit expression in CD44^+^c-Kit^+^ cells. **(D)**. pseudo-color plots showing B220 and CD3ε expression in total splenocytes (left) and TCRβ and γδTCR expression in CD3ε^+^ splenocytes (middle) and CD4 and CD8α expression in CD3ε^+^ TCRβ^+^ splenocytes (right). **(E)**. Contour plots showing B220 and CD3ε expression on cells in peripheral lymph nodes (left) and CD4 and CD8α expression in CD3ε^+^ cells (middle). Expression of ThPOK in cells in indicated gate with arrow is shown as shaded histograms with Thpok expression in *Bcl11b^+/+^* CD8α^+^ cells as an open histogram.

To further examine T cell development at later stages from *Bcl11b^N797K/N797^* progenitors, we transplanted fetal liver (FL) cells prepared from *Bcl11b^+/+^* and *Bcl11b^N797K/N797K^* at 13.5- or 14.5-dpc embryos into lethally irradiated *Rag1*-deficient recipients (**Supplementary Figure 3E**). Eight weeks after transplantation, we easily detected CD3ε^+^ cells in the peripheral blood of the recipients injected with *Bcl11b^+/+^* progenitors. In contrast, very few CD3ε^+^ cells were in the two recipients injected with *Bcl11b^N797K/N797K^* progenitors, whereas their CD19^+^ B lymphocytes were well-reconstituted (**Supplementary Figure 3E**). The emergence of any CD3ε^+^ cells in the peripheral blood suggested that *Bcl11b^N797K/N797K^* progenitors might retain some potency to give rise to mature T cells, prompting us to examine the lymphoid tissues. In the thymus of two recipients (#1 and #2), CD4^+^CD8^+^ DP thymocytes were reconstituted to some extent; however, as expected, the majority of CD4^−^CD8^+^ thymocytes were CD24^+^ ISP8 thymocytes (**Figure 4A**). Only a few mature CD24^−^TCRβ^+^ thymocytes and aberrant CD4^−^CD8α^−^ mature thymocytes emerged in these thymi (**Figure 4B**). In the CD4^−^CD8^−^ DN thymocyte population, the frequency of CD44^+^CD25^+^ and c-Kit^+^CD44^+^ cells was higher in recipients injected with *Bcl11b^N797K/N797K^* progenitors than those injected with *Bcl11b^+/+^* progenitors (**Figure 4C**). In the c-Kit^+^CD44^+^ population, the frequency of CD25^−^ cells. which corresponds to ETP, was clearly higher in both recipients. Thus, engraftment occurred but was blocked in T-lineage development by the Bcl11b mutation.

In the spleen, the frequency of CD3ε^+^ T cells was approximately 1% in recipients injected with *Bcl11b^N797K/N797K^*, while CD3ε^+^ T cells were reconstituted in more than 20% of *Bcl11b^+/+^* progenitors (**Figure 4D**). Among the CD3ε^+^ T cells generated from *Bcl11b^N797K/N797K^* progenitors, γδTCR^+^ cells were almost undetectable. This finding indicates that the potency to generate T cells was not completely lost in *Bcl11b^N797K/N797K^* progenitors. Interestingly, *Bcl11b^N797K/N797K^* αβT cells showed dominant differentiation into CD4^+^ T cells with the emergence of CD4^−^CD8α^−^ αβT cells. Thus, *Bcl11b^N797K/N797K^* progenitors failed to generate CD8^+^ T cells. Given that Bcl11b was shown to regulate the *Zbtb7b* gene, which encodes the CD4 helper-lineage specifying transcription factor ThPOK, we examined ThPOK expression via flow cytometry. We found that ThPOK expression was down-regulated not only in CD4^−^CD8^−^ cells but also in CD4^+^CD8^−^ αβT cells compared to *Bcl11b^+/+^* CD4^+^CD8^−^ αβT cells (**Figure 4E**). Therefore, the lack of CD8 SP mature cells was not because of overexpression of ThPOK.

### *Bcl11b^N797K/N797K^* progenitors showed impaired T cell development from in OP9/DL1 culture

**Figure 5:**
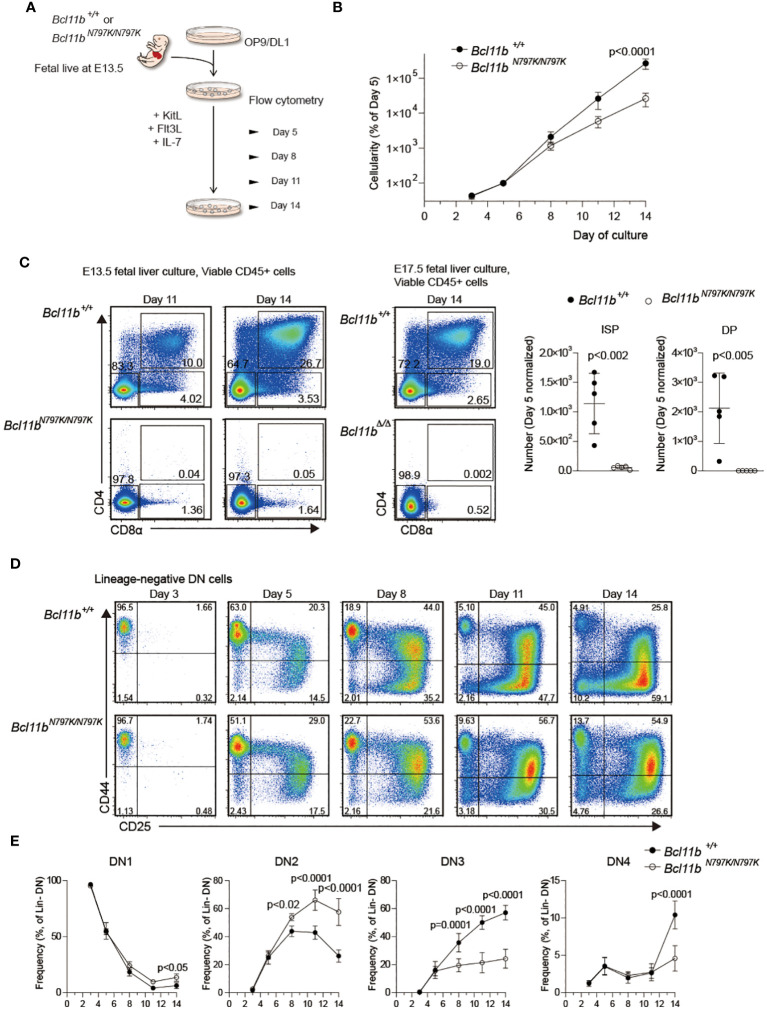
Impaired T cell development from *Bcl11b^N197K/N797K^* progenitors in culture. **(A)**. A scheme showing an experimental flow for inducing T cell differentiation from E13.5 FL on OP9-DLL1 stromal cells. **(B)**. Number of cells was assessed at the indicated timepoints, and was normalized to day 5. **(C)**. pseudo-color plots showing expression of CD4 and CD8α on cells differentiated from *Bcl11b^+/+^* and *Bcl11b^N797K/N797K^* 13.5 dpc FL cells at day 11 and 14 of culture. As for reference, expression of CD4 and CD8α on cells differentiated from *Bcl11b^+/+^* and *Bcl11b^Δ/Δ^* 17.5 dpc FL cells at day 14 of culture were shown at right. Graphs showing quantification of ISP8 and CD4^+^CD8α^+^ DP cells at day14. **(D)**. Representative pseudo-color plots showing expression of CD25 and CD44 by lineage-negative CD4^−^CD8α^−^ DN cells differentiated from Bcl11b^+/+^ and *Bcl11b^N797K/N797K^* 13.5 dpc FL cell sat the indicated timepoints. **(E)**. Graphs showing the frequency of CD44^+^CD25^−^ (DN1), CD44^+^CD25^+^ (DN2), CD44^−^CD25^+^ (DN3) and CD44^−^CD25^−^ (DN4) subset. Result pooled from two independent experiments with cells from five donors of each genotype. Graphs in B and E show Mean ± SEM and significance tested with two way ANOVA and Sidak’s correction, Graphs in B show Mean ± SD, significance tested with Student’s two-tailed *t* test.

A decline in CD45.1 frequency at the DN1 to DN2 transition in the competitive repopulation assay and an increase in the ETP frequency in *Rag1*-deficient recipients suggest that a developmental block occurs at the this transition in the presence of the Bcl11b^N797K^ mutant. To address this point further, we performed an *in vitro* T cell development assay using FL cells and OP9 stroma cells expressing a Notch ligand, delta-like 1 (OP9-DL1) (**Figure 5A**). Lineage-depleted 13.5-dpc FL cells were cultured on OP9-DL1 cells with supplement of cytokines, under conditions that support T-lineage development. Culture cellularities were calculated at all observation days, and frequencies of hemotopoetic cells were normalized to day 5 values to allow averaging across samples. The expansion of *Bcl11b^N797K/N797K^* cells was slower than that of *Bcl11b^+/+^* cells (**Figure 5B**). CD4^+^CD8^+^ cells emerged from *Bcl11b^+/+^* cells on day 11, and the frequency of CD4^+^CD8^+^ cells increased by approximately 30% by day 14. On the contrary, no CD4^+^CD8^+^ cells emerged from *Bcl11b^N797K/N797K^* cells, even at day 14, despite the appearance of a small but significant CD4^−^CD8^+^ subset. As a reference, we also cultured *Bcl11b^Δ/Δ^* 17.5-dpc FL cells and found that the differentiation of CD4^−^CD8^+^ cells was almost undetected (**Figure 5C**). The higher potency of T cell development observed in *Bcl11b^N797K/N797K^* cells than *Bcl11b^Δ/Δ^* cells in culture is in line with the results obtained in recipient mice. Comparison of the frequency of DN1, DN2, DN3 and DN4 subsets during culture revealed a higher frequency of *Bcl11b^N797K/N797K^* DN2 subset and a lower DN3 frequency from day 8 to day14 than *Bcl11b^+/+^* cells, indicating a developmental block at transition from DN2 to DN3 in *Bcl11b^N797K/N797K^* cells. Interestingly, the frequency of the DN1 subset slightly increased from days 11 to 14 and was higher in *Bcl11b^N797K/N797K^* cell cultures than *Bcl11b^+/+^* cells.

### Emergence of c-Kit^+^CD25^−^CD44^−^ and c-Kit^−^CD25^−^CD44^+^ cells from Bcl11b^N797K/N797K^ DN2 cells

**Figure 6:**
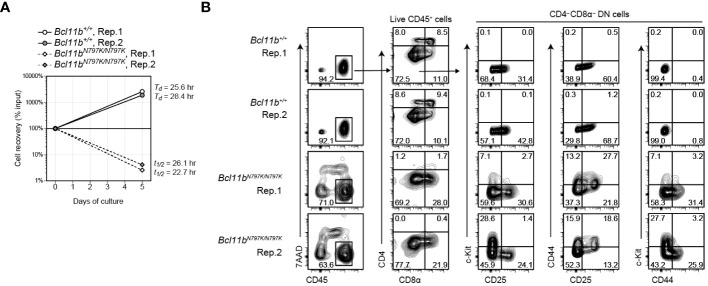
Emergence of CD44^+^ and c-Kit^+^ cells from *Bcl11b^N797K/N797K^* DN2 cells in culture. **(A)**. Graph showing a cell recovery rate of *Bcl11b^+/+^* and *Bcl11b^N797K/N797K^* DN2 cells at day5 in culture on TSt-4/Dll4 cells. *T_d_* and *T_1/2_* are calculated doubling and half time, respectively. **(B)**. Left plots showing expression of CD45 and 7-AAD incorporation to define live CD45^+^ cells at day 5 of *Bcl11b^+/+^* and *Bcl11b^N797K/N797K^* DN2 cells culture. CD4 and CD8α on total CD45^+^ cells and CD25, CD44 and c-Kit expression on CD4^−^CD8α^−^ DN cells are shown.

Considering the stepwise development of T cells from DN1 to DN2, DN2 to DN3 and DN3 to DN4, an increase in the CD44^+^CD25^−^ DN1 frequency in the later stage of *Bcl11b^N797K/N797K^* culture was unusual but consistent with the higher frequency of CD44^+^CD25^−^c-Kit^+^ DN1-like cells observed in *Rag1*-defcient recipients. Given that *Bcl11b^N797K/N797K^* DN2 cells showed a developmental arrest upon transition to DN3 cells, we examined the differentiation pathway of DN2 cells. To this aim, CD44^+^CD25^+^ DN2 cells were purified from *Rag1*-defcient recipients injected with either *Bcl11b^+/+^* or *Bcl11b^N797K/N797K^* FL cells and cultured on TSt-4/Dll4 feeder cells. While *Bcl11b^+/+^* DN2 cells easily expanded and showed a 10-fold increase in their numbers within 5 days, most *Bcl11b^N797K/N797K^* DN2 cells were dying, and less than 5% of the cells remained alive for 5 days in culture (**Figure 6A**). Flow cytometry analysis of living CD45^+^ cells showed the differentiation of CD4^+^CD8α^+^ DP cells from *Bcl11b^+/+^* but not *Bcl11b^N797K/N797K^* DN2 cells. Interestingly, in the CD4^−^CD8α^−^ DN population, c-Kit^+^ cells emerged from *Bcl11b^N797K/N797K^* cells but not *Bcl11b^+/+^* DN2 cells. Most c-Kit^+^ cells were negative for CD44 expression. CD44^+^ cells also emerged specifically from *Bcl11b^N797K/N797K^* DN2 cells, and less than half of these CD44^+^ cells lost CD25 expression. Thus, c-Kit^+^CD44^−^ and c-Kit^−^CD44^+^ cells differentiated from *Bcl11b^N797K/N797K^* DN2 cells at least in our culture condition.

### The Bcl11b^N797K^ mutant protein inhibits Cd4 gene expression

**Figure 7:**
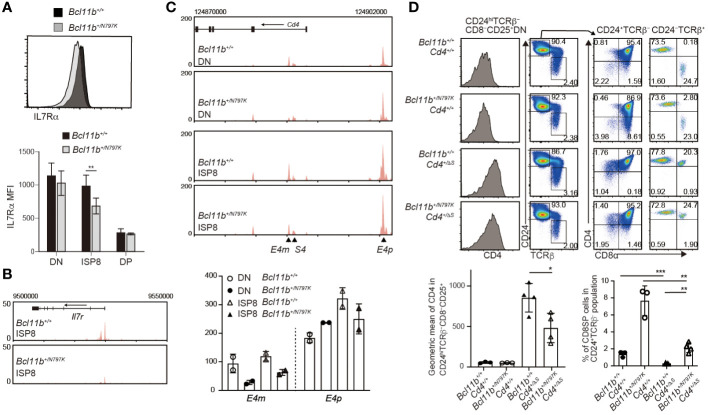
Increase of immature CD8SP thymocytes is mediated in part by impaired *Cd4* gene activation. **(A)**. Representative histogram showing IL-7Rα expression in immature CD8 SP thymocytes of *Bcl11b^+/+^* (black) and *Bcl11b^+/N797K^* (gray) mice. Lower graph shows mean fluorescent intensity (MFI) of IL-7Rα expression in CD4^−^CD8α^−^ DN, ISP8 and CD4^−^CD8α^−^ DP thymocytes. **(B)**. ATAC-seq tracks at the *Il7r* locus in ISP8 thymocytes of *Bcl11b^+/+^* and *Bcl11b^+/N797K^* mice. One of duplicated samples are shown. **(C)**. ATAC-seq tracks at the *Cd4* locus in CD4^−^CD8α^−^ DN and ISP8 thymocytes of *Bcl11b^+/+^* and *Bcl11b^+/N797K^* mice. Positions of *E4p*, *E4m* and *S4* are indicated with arrowhead. One of duplicated samples are shown. Lower graph shows RPKM at the *E4p* (chr6:124900510) and *E4m* (chr6:124885330) of duplicated DN and ISP8 cells of *Bcl11b^+/+^* and *Bcl11b^+/N797K^* mice. **(D)**. Representative pseudo-color plots showing CD24 and TCRβ expression in expressions in total thymocytes and CD4 and CD8α expression in CD24^+^TCRβ^−^ and CD24^−^TCRβ^+^ thymocytes of *Bcl11b^+/+^: Cd4^+/+^*, *Bcl11b^+/N797K^: Cd4^+/+^, Bcl11b^+/+^: Cd4^+/ΔS^* and *Bcl11b^+/N797K^: Cd4^+/ΔS^* mice. Left histograms showing CD4 expression in CD24^hi^TCRβ^−^CD8^−^CD25^+^ cells. Lower right graph showing a frequency of CD4^−^CD8^+^ SP cells in CD24^+^TCRβ^−^ population of mice with indicated genotype. Lower left graph showing a geographic mean of CD4 expression in CD24^hi^TCRβ^−^CD8^−^CD25^+^ thymocytes of mice with indicated genotype. Unpaired t-tests were performed. *: p < 0.05, **: p < 0.005, ***: p < 0.0005.

Our results showed an increase in the frequency of ISP8 thymocytes by the *Bcl11b^N797K^* variant protein. There are at least two mechanisms that lead to an increase in the ISP8 subset: a developmental block from the ISP8 to the CD4^+^CD8^+^ DP transition, or the attenuated activation of *Cd4* gene, which causes CD4 downregulation in DP thymocytes. In order to characterize the ISP8 thymocytes that emerged in *Bcl11b^+/N797K^* mice, we examined the expression of IL-7Rα, which was downregulated during the differentiation of CD4^−^CD8α^−^ DN into CD4^+^CD8α^+^ DP thymocytes with reduced chromatin accessibility at the *Il7r* promoter region, where Bcl11b binding was detected using ChIP-seq (**Supplementary Figure 4A, B**). Interestingly, both IL-7Rα expression level and chromatin accessibility at the *Il7r* promoter loci were lower in *Bcl11b^+/N797K^* ISP8 cells than in *Bcl11b^+/+^* ISP8 cells (**Figures 7A, B**). In addition, Rorγt expression, which was induced from DN to ISP8 transition and slightly declined in DP thymocytes, was lower in *Bcl11b^+/N797K^* ISP8 cells than in *Bcl11b^+/+^* ISP8 cells, and was similar between ISP8 and CD4^+^CD8^+^ DP thymocytes in *Bcl11b^+/N797K^* mice (**Supplementary Figure 4C**). Thus, regarding *Il7r* and *Rorc* gene regulation, *Bcl11b^+/N797K^* ISP8 cells showed characteristics similar to CD4^+^CD8α^+^ DP thymocytes, suggesting that impaired CD4 induction is involved in the accumulation of ISP8 cells in *Bcl11b^+/N797K^* mice. Regarding *Cd4* gene regulation, two transcriptional enhancers, proximal enhancer (*E4p*) (28, 29) and maturation enhancer (*E4m*) (24, 30), have been identified in the *Cd4* locus. Genetic ablation of the *E4p* from the *Cd4* locus showed that *E4p* is essential for inducing CD4 expression at the transition from CD4^−^CD8^−^ DN to CD4^+^CD8^+^ DP thymocytes (29). ATAC-seq results showed increased chromatin accessibility at both *E4p* and *E4m* regions during the DN to DP transition, where Bcll1b binding was also detected by ChIP-seq (**Supplementary Figure 4D**). Interestingly, chromatin accessibility in the *E4m* region was lower in *Bcl11b^+/N797K^* ISP8 cells than in *Bcl11b^+/+^* ISP8 cells, while chromatin accessibility at the *E4p* region was comparable between these cells (**Figure 7C**).

The expression of the *Cd4* gene was repressed by the intronic *Cd4* silencer (*S4*) in both CD4^−^CD8^−^ DN and mature CD4^−^CD8^+^ SP thymocytes (31). The removal of the *S4* from the *Cd4* locus, referred to as the *Cd4^+/ΔS^* allele (24), resulted in the de-repression of CD4 from CD25^+^ DN2/3 thymocytes and accelerated the CD4 upregulation at the transition from the DN to DP stage (32). Thus, CD4 induction at the DN-to-DP transition is regulated by both the activation of *E4p* and the inactivation of *S4*. To examine whether and to what extent the removal of the *S4* reverses the increase in the ISP8 subset in *Bcl11b^+/N797K^* mice, we generated *Bcl11b^+/N797K^:Cd4^+/ΔS^* double-mutant mice. While the removal of *S4* reduced the frequency of CD4^−^CD8^+^ thymocytes in the CD24^+^TCRβ^−^ population to a nearly undetectable level in the *Bcl11b^+/+^* background, a small proportion of CD4^−^CD8^+^ thymocytes was still detected in *Bcl11b^+/N797K^: Cd4^+/ΔS^* mice. Thus, the genetic ablation of *S4* did not completely reverse the increase in ISP8 cell frequency. This result suggests that the Bcl11b^N797K^ mutant inhibits *Cd4* gene activation by affecting enhancer activity at the DN-DP transition stage or from an earlier stage. Given that *Bcl11b* gene is expressed at the DN2 stage and CD4 de-repression by loss of the *S4* is induced in CD25^+^ DN cells, we examined CD4 expression levels in CD25^+^ DN cells and found that it was lower in *Bcl11b^+/N797K^: Cd4^+/ΔS^* mice than *Bcl11b^+/+^: Cd4^+/ΔS^* mice. Given that *E4p* is functionally responsible for CD4 induction in DP thymocytes, the Bcl11b^N797^ mutant may interfere with the function of *E4p* in CD25^+^ DN cells. However, it is noteworthy that chromatin accessibility in DN thymocytes was reduced by the Bcl11b^N797K^ protein only at the *E4m* region, not at the *E4p* region (**Figure 7C**). This suggests that Bcl11b is involved in a step-wise activation of *E4p* enhancer after the chromatin structure is altered and that N797K replacement may attenuate such Bcl11b function.

## Discussion

In this study, we examined how the Bcl11b^N797K^ mutant protein impaired T cell development in mice, corresponding to the human BCL11B^N807K^ variant isolated from a patient with impaired T cell development. As observed in human patients, T cell and ILC2 development was impaired in *Bcl11b^+/N797K^* mice, indicating that the pathogenesis of *BCL11B^+/N807K^* patients was recapitulated by the *Bcl11b^N797K^* mutation in mice, at least, to some extent.

One apparent phenotype caused by the Bcl11b^N797K^ mutant protein during early T cell development is an increase in ISP8 thymocyte frequency. Previous studies using germline *Bcl11b*-deficient mice showed that the loss of Bcl11b causes developmental arrest at the DN3 stage. The inactivation of *Bcl11b* gene by Cre-mediated site-specific recombination between loxP sites revealed that Bcl11b functions also at later T cell developmental stages. For instance, the inactivation of *Bcl11b* at the DP stage by the *Cd4-Cre* transgene revealed its essential role during positive selection (8) and iNKT cell development (12). However, owing to the lack of an appropriate Cre transgenic line that expresses Cre at the late DN3 or DN4 stages, it has been difficult to determine the function of Bc111b at the DN-to-DP transition. We have previously reported that mice homozygous for the mutant *Bcl11b^m^* allele, which produces a truncated Bcl11b protein lacking the last ZnF domain showed accumulation of an immature ISP8 subset in P0 neonates. However, analyses at P0 stage alone are insufficient to exclude the possibility that an increase in ISP8 frequency stems from a delay in T cell ontogeny. In the present study, we observed an increase in ISP8 frequency in adult *Bcl11b^+/N979K^* mice. In addition, in the recipients of mixed bone marrow chimera, there was a sharp increase in the ratio of CD45.2^+^ *Bcl11b^+/N979K^* to CD45.1^+^ *Bcl11b^+/+^* cells at the ISP8 stage in recipients of mixed bone marrow chimera. These findings indicate that the appropriate differentiation of DN thymocytes into DP thymocytes is regulated by a Bcl11b-dependent cell intrinsic mechanism. It was previously shown that the mis-sense *Ikzf3* variant encoding the Aiolos^G158R^ mutant caused a B cell deficiency by interfering with Ikaros function when Aiolos^G158R^ makes heterodimer with Ikaros (33), a mechanism referred to as “heterodimeric interference”. Therefore, although the accumulation of ISP8 cells occurs via a cell-autonomous mechanism, it remains unclear whether the loss of Bcl11b function itself or an interference with proteins functions other than Bcl11b is responsible for this phenotype. However, by analyzing the effects of the rapid loss of the Bcl11b protein in mice using the auxin-inducible degron 2 (AID2) system (34), we also observed an accumulation of the ISP8 subset within two days after a loss of Bcl11b protein (manuscript in preparation). This observation supports the idea that Bcl11b function itself is important for the proper differentiation of DN thymocytes into DP thymocytes.

Mechanistically, we showed that *Cd4* gene activation was attenuated by the Bcl11b^N797K^ mutant protein. Bcl11b binds to three major regulatory regions in the *Cd4* locus, *E4p*, *E4m*, and *S4* (24). By analyzing the combined mutation of the *E4p* deletion (*Cd4^ΔE4p^*) with the loss of Bcl11b in DP thymocytes (*Bcl11b^F/F^: Cd4-Cre*), Bcl11b was proposed to be important for *E4m* function after positive selection (24). In this study, chromatin accessibility in the *E4m* region was lower in *Bcl11b^+/N979K^* DN and ISP8 cells, in which the *E4m* enhancer had not yet become functional. However, the lower CD4 expression in CD25^+^ DN2/3 cells of *Bcl11b^+/N797^: Cd4^+/ΔS^* mice than in *Bcl11b^+/+^: Cd4^+/ΔS^* mice suggests that *E4p* function in DN thymocytes could be impaired by the Bcl11b^N979K^ mutant. However, chromatin accessibility in the *E4p* region was not affected by Bcl11b^N797K^ protein. In many cases of enhancer activation, the opening of chromatin structures by pioneering factors proceeds before enhancer activation (35). Taken together, these observations suggest that Bcl11b regulates *E4p* activity via recruiting transcriptional activators. On the other hand, Bcl11b regulates *E4m* activity not only by recruiting transcriptional activators at later stage, such as in DP thymocytes, but also by altering the chromatin structure at an early stage in DN thymocytes.

In this study, we observed that *Bcl11b^N797K/N797K^* CD25^+^CD44^+^ DN2 cells give rise to c-Kit^+^CD25^−^CD44^−^ and c-Kit^−^CD25^−^CD44^+^ cells in culture. This is consistent with the increase in the number of CD25^−^CD44^+^ cells from days 11 to day 14 in the culture of 14.5-dpc *Bcl11b^N797K/N797K^* FL cells on OP9-DLL1 stromal cells. Although the natures of these c-Kit^+^CD25^−^CD44^−^ and c-Kit^−^CD25^−^CD44^+^ cells were not characterized in this study, these cells could differ from authentic c-Kit^+^CD25^−^CD44^+^ ETPs. In this regard, the emergence of c-Kit^+^CD25^−^CD44^−^ and c-Kit^−^CD25^−^CD44^+^ cells was not caused by de-differentiation of CD25^+^CD44^+^ DN2 cells. Rather, *Bcl11b^N797K/N797K^* DN2 cells failed to enter the proper developmental pathway for the T-lineage, and *Kit* and *Cd44* are expressed in aberrant differentiation pathway. Further studies will unravel the characteristics of these c-Kit^+^CD25^−^CD44^−^ and c-Kit^−^CD25^−^CD44^+^ cells.

Our study also provides insights into the functional domains of Bcl11b. Among its six C_2_H_2_-type ZnF domains, the second and third have been proposed to mediate DNA binding (2). A previous study reported a patient with severe T cell deficiency harboring a *de novo BCL11B* heterozygous variant, causing a replacement of the N441 residue within the second ZnF with a K, resulting in the BCL11B^N441K^ variant protein (15). Thus, this N to K replacement happened to be introduced at the middle and the C-terminal C_2_H_2_ ZnF domain in different patients showing similar T cell deficiency, although the position of N relative to the second C is different; N^441^ is the 9^th^ and N^807^ is the 6^th^. We also generated a mutant mouse strain, *Bcl11b^N440K^*, which produced a murine Bcl11b^N440K^ protein corresponding to human BCL11B^N441K^. Compared with *Bcl11b^+/N797K^* mice, *Bcl11b^+/N440K^* mice showed more severer growth retardation and died at approximately three weeks of age (manuscript submitted). Although T cell development was delayed also in *Bcl11b^+/N440K^* mice, we did not observe an apparent accumulation of ISP8 cells. Thus, the phenotypes caused by Bcl11b^N440K^ and Bcl11b^N797K^ mutant protein were not the same. Given the growth retardation and impaired T cell development in both *Bcl11b^+/N440K^* and *Bcl11b^+/N797K^* mice, both Bcl11b^N440K^ and Bcl11b^N797K^ mutants function in a dominant negative form; however, the mode of action of these proteins is different.

In addition to the impaired differentiation of conventional T cells, the Bcl11b^N797K^ mutant protein also affects the differentiation of other immune cells. The development of iNKT cells was severely inhibited in *Bcl11b^+/N797K^* mice in an intrinsic T cell-dependent manner. Interestingly, the reduction of iNKT cells was milder in *Bcl11b^+/N440K^* than in *Bcl11b^+/N797K^* mice. The competitive reconstitution assay clearly showed that the developmental capacity of *Bcl11b^+/N797K^* progenitors to become γδT cells was outcompeted by control cells. Since the first manuscript of *Bcl11b*-deficient mice did not show a clear defect in thymic γδT cell development (4), the roles of Bcl1b in regulating γδT cells has not been well examined. It would be interesting to compare the capacity of γδT cells to differeniciate between *Bcl11b^+/N797K^* and *Bcl11b^+/Δ^* or *Bcl11^N797K/N797K^* and *Bcl11b^Δ/Δ^* progenitors. If *Bcl11b^+/N797K^* progenitors are outcompeted by *Bcl11b^+/Δ^* progenitors, the Bcl11b^N797K^ mutant protein interferes with the function of molecules other than Bcl11b. In addition to T-lineage cells, *Bcl11b^+/N797K^* mice also showed impaired ILC2 development, which was also observed in human *BCL11B^+/N807K^* patients. Unraveling the molecular mechanisms by which the Bcl11b^N797K^ mutant protein inhibits the differentiation of iNKT, γδT and ILC2 cells await future studies.

In summary, we characterized the immune phenotypes and esulting pathogeneses caused by the human patient-derived BCL11B mutant protein, BCL11B^N807K^, using s mouse model. We also elucidated the novel role of Bcl11b in efficiently activating the *Cd4* gene at the DP transition. Although other variants of the *BCL11B* gene have been isolated from patients with IEIs, the function of these mutant BCL11B proteins have not been examined in a mouse model. Hence, generating mouse models for each variant is a powerful tool to determine whether each *BCL11B* mutation has a different impact on immune cell developments.

## Data availability statement

The datasets presented in this study can be found in online repositories. The names of the repository/repositories and accession number(s) can be found below: SRP473952 (SRA).

## Ethics statement

The animal study was approved by The Institutional Animal Care and Use Committee (IACUC) of RIKEN Yokohama Branch. The study was conducted in accordance with the local legislation and institutional requirements.

## Author contributions

KM: Formal analysis, Investigation, Writing – review & editing. kO: Formal analysis, Investigation, Writing – review & editing. TS: Investigation, Writing – review & editing. MY: Investigation, Writing – review & editing. TE: Writing – review & editing, Data curation, Formal analysis. NS: Investigation, Writing – review & editing. HO: Formal analysis, Investigation, Writing – review & editing. TM: Investigation, Supervision, Writing – review & editing. ER: Investigation, Writing – review & editing. IT: Conceptualization, Resources, Supervision, Writing – original draft, Writing – review & editing.

## References

[1] Tangye SG Al-Herz W Bousfiha A Cunningham-Rundles C Franco JL Holland SM. Human inborn errors of immunity: 2022 update on the classification from the international union of immunological societies expert committee. J Clin Immunol. (2022) 42:1473–507. doi: 10.1007/s10875-022-01289-3 PMC9244088 35748970

[2] Avram D Fields A Senawong T Topark-Ngarm A Leid M. COUP-TF (chicken ovalbumin upstream promoter transcription factor)-interacting protein 1 (CTIP1) is a sequence-specific DNA binding protein. Biochem J. (2002) 368:555–63. doi: 10.1042/bj20020496 PMC1223006 12196208

[3] Liu P Keller JR Ortiz M Tessarollo L Rachel RA Nakamura T. Bcl11a is essential for normal lymphoid development. Nat Immunol. (2003) 4:525–32. doi: 10.1038/ni925 12717432

[4] Wakabayashi Y Watanabe H Inoue J Takeda N Sakata J Mishima Y. Bcl11b is required for differentiation and survival of alphabeta T lymphocytes. Nat Immunol. (2003) 4:533–9. doi: 10.1038/ni927 12717433

[5] Li L Leid M Rothenberg EV. An early T cell lineage commitment checkpoint dependent on the transcription factor Bcl11b. Science. (2010) 329:89–93. doi: 10.1126/science.1188989 20595614 PMC2935300

[6] Ikawa T Hirose S Masuda K Kakugawa K Satoh R Shibano-Satoh A. An essential developmental checkpoint for production of the T cell lineage. Science. (2010) 329:93–6. doi: 10.1126/science.1188995 20595615

[7] Li P Burke S Wang J Chen X Ortiz M Lee S-C. Reprogramming of T cells to natural killer-like cells upon Bcl11b deletion. Science. (2010) 329:85–9. doi: 10.1126/science.1188063 PMC3628452 20538915

[8] Albu DI Feng D Bhattacharya D Jenkins NA Copeland NG Liu P. BCL11B is required for positive selection and survival of double-positive thymocytes. J Exp Med. (2007) 204:3003–15. doi: 10.1084/jem.20070863 PMC2118514 17998389

[9] Kojo S Tanaka H Endo TA Muroi S Liu Y Seo W. Priming of lineage-specifying genes by Bcl11b is required for lineage choice in post-selection thymocytes. Nat Commun. (2017) 8:702. doi: 10.1038/s41467-017-00768-1 28951542 PMC5615048

[10] Vanvalkenburgh J Albu DI Bapanpally C Casanova S Califano D Jones DM. Critical role of Bcl11b in suppressor function of T regulatory cells and prevention of inflammatory bowel disease. J Exp Med. (2011) 208:2069–81. doi: 10.1084/jem.20102683 PMC3182057 21875956

[11] Hasan SN Sharma A Ghosh S Hong S-W Roy-Chowdhuri S Im S-H. Bcl11b prevents catastrophic autoimmunity by controlling multiple aspects of a regulatory T cell gene expression program. Sci Adv. (2019) 5:eaaw0706. doi: 10.1126/sciadv.aaw0706 31457081 PMC6685721

[12] Albu DI VanValkenburgh J Morin N Califano D Jenkins NA Copeland NG. Transcription factor Bcl11b controls selection of invariant natural killer T-cells by regulating glycolipid presentation in double-positive thymocytes. Proc Natl Acad Sci U S A. (2011) 108:6211–6. doi: 10.1073/pnas.1014304108 PMC3076841 21444811

[13] Walker JA Oliphant CJ Englezakis A Yu Y Clare S Rodewald H-R. Bcl11b is essential for group 2 innate lymphoid cell development. J Exp Med. (2015) 212:875–82. doi: 10.1084/jem.20142224 PMC4451131 25964370

[14] Yu Y Wang C Clare S Wang J Lee S-C Brandt C. The transcription factor Bcl11b is specifically expressed in group 2 innate lymphoid cells and is essential for their development. J Exp Med. (2015) 212:865–74. doi: 10.1084/jem.20142318 PMC4451136 25964371

[15] Punwani D Zhang Y Yu J Cowan MJ Rana S Kwan A. Multisystem anomalies in severe combined immunodeficiency with mutant BCL11B. N Engl J Med. (2016) 375:2165–76. doi: 10.1056/NEJMoa1509164 PMC5215776 27959755

[16] Lessel D Gehbauer C Bramswig NC Schluth-Bolard C Venkataramanappa S Gassen van KLI. BCL11B mutations in patients affected by a neurodevelopmental disorder with reduced type 2 innate lymphoid cells. Brain. (2018) 141:2299–311. doi: 10.1093/brain/awy173 PMC6061686 29985992

[17] Homma TK Freire BL Honjo Kawahira RS Dauber A Funari Assis MF Lerario AM. Genetic disorders in prenatal onset syndromic short stature identified by exome sequencing. J Pediatr. (2019) 215:192–8. doi: 10.1016/j.jpeds.2019.08.024 31630891

[18] Prasad M Balci TB Prasad C Andrews JD Lee R Jurkiewicz MT. BCL11B-related disorder in two canadian children: Expanding the clinical phenotype. Eur J Med Genet. (2020) 63:104007. doi: 10.1016/j.ejmg.2020.104007 32659295

[19] Qiao F Wang C Luo C Wang Y Shao B Tan J. A *De Novo* heterozygous frameshift mutation identified in BCL11B causes neurodevelopmental disorder by whole exome sequencing. Mol Genet Genomic Med. (2019) 7:e897. doi: 10.1002/mgg3.897 31347296 PMC6732278

[20] Yang S Kang Q Hou Y Wang L Li L Liu S. Mutant BCL11B in a patient with a neurodevelopmental disorder and T-cell abnormalities. Front Pediatr. (2020) 8:544894. doi: 10.3389/fped.2020.544894 33194885 PMC7641641

[21] Zhao X Wu B Chen H Zhang P Qian Y Peng X. Case report: A novel truncating variant of BCL11B associated with rare feature of craniosynostosis and global developmental delay. Front Pediatr. (2022) 10:982361. doi: 10.3389/fped.2022.982361 36275064 PMC9582536

[22] Lu HY Sertori R Contreras AV Hamer M Messing M Bel Del KL. A novel germline heterozygous BCL11B variant causing severe atopic disease and immune dysregulation. Front Immunol. (2021) 12:788278. doi: 10.3389/fimmu.2021.788278 34887873 PMC8650153

[23] Eto K Machida O Yanagishita T Yamamoto KS Chiba K Aihara Y. Novel BCL11B truncation variant in a patient with developmental delay, distinctive features, and early craniosynostosis. Hum Genome Var. (2022) 9:43. doi: 10.1038/s41439-022-00220-x 36470856 PMC9722650

[24] Kojo S Yasmin N Muroi S Tenno M Taniuchi I. Runx-dependent and silencer-independent repression of a maturation enhancer in the Cd4 gene. Nat Commun. (2018) 9:3593. doi: 10.1038/s41467-018-05803-3 30185787 PMC6125603

[25] Stritesky GL Jameson SC Hogquist KA. Selection of self-reactive T cells in the thymus. Annu Rev Immunol. (2012) 30:95–114. doi: 10.1146/annurev-immunol-020711-075035 22149933 PMC3518413

[26] Sabbagh Q Haghshenas S Piard J Trouvé C Amiel J Attié-Bitach T. Clinico-biological refinement of BCL11B-related disorder and identification of an episignature: a series of 20 unreported individuals. Genet Med. (2024) 2023:101007. doi: 10.1016/j.gim.2023.101007 37860968

[27] Arlotta P Molyneaux BJ Chen J Inoue J Kominami R Macklis JD. Neuronal subtype-specific genes that control corticospinal motor neuron development. vivo. Neuron. (2005) 45:207–21. doi: 10.1016/j.neuron.2004.12.036 15664173

[28] Sawada S Littman DR. Identification and characterization of a T-cell-specific enhancer adjacent to the murine CD4 gene. Mol Cell Biol. (1991) 11:5506–15. doi: 10.1128/MCB.11.11.5506 PMC361920 1922061

[29] Chong MM Simpson N Ciofani M Chen G Collins A Littman DR. Epigenetic propagation of CD4 expression is established by the Cd4 proximal enhancer in helper T cells. Genes Dev. (2010) 24:659–69. doi: 10.1101/gad.1901610 PMC2849123 20360383

[30] Issuree PD Day K Au C Raviram R Zappile P Skok JA. Stage-specific epigenetic regulation of CD4 expression by coordinated enhancer elements during T cell development. Nat Commun. (2018) 9:3594. doi: 10.1038/s41467-018-05834-w 30185805 PMC6125341

[31] Sawada S Scarborough JD Killeen N Littman DR. A lineage-specific transcriptional silencer regulates CD4 gene expression during T lymphocyte development. Cell. (1994) 77:917–29. doi: 10.1016/0092-8674(94)90140-6 8004678

[32] Taniuchi I Sunshine MJ Festenstein R Littman DR. Evidence for distinct CD4 silencer functions at different stages of thymocyte differentiation. Mol Cell. (2002) 10:1083–96. doi: 10.1016/S1097-2765(02)00735-9 12453416

[33] Yamashita M Kuehn HS Okuyama K Okada S Inoue Y Mitsuiki N. A variant in human AIOLOS impairs adaptive immunity by interfering with IKAROS. Nat Immunol. (2021) 22:893–903. doi: 10.1038/s41590-021-00951-z 34155405 PMC8958960

[34] Yesbolatova A Saito Y Kitamoto N Makino-Itou H Ajima R Nakano R. The auxin-inducible degron 2 technology provides sharp degradation control in yeast, mammalian cells, and mice. Nat Commun. (2020) 11:5701. doi: 10.1038/s41467-020-19532-z 33177522 PMC7659001

[35] Thurman RE Rynes E Humbert R Vierstra J Maurano MT Haugen E. The accessible chromatin landscape of the human genome. Nature. (2012) 489:75–82. doi: 10.1038/nature11232 22955617 PMC3721348

