# Supplementary material for: A Bcl11bN797K variant isolated from an immunodeficient patient inhibits early thymocyte development in mice
Source: Front Immunol. 2024 Mar 1;15:1363704. doi: 10.3389/fimmu.2024.1363704 (PMC10940544; doi:10.3389/fimmu.2024.1363704)
Supplement: Supplementary file 1 [file DataSheet_1.pdf]

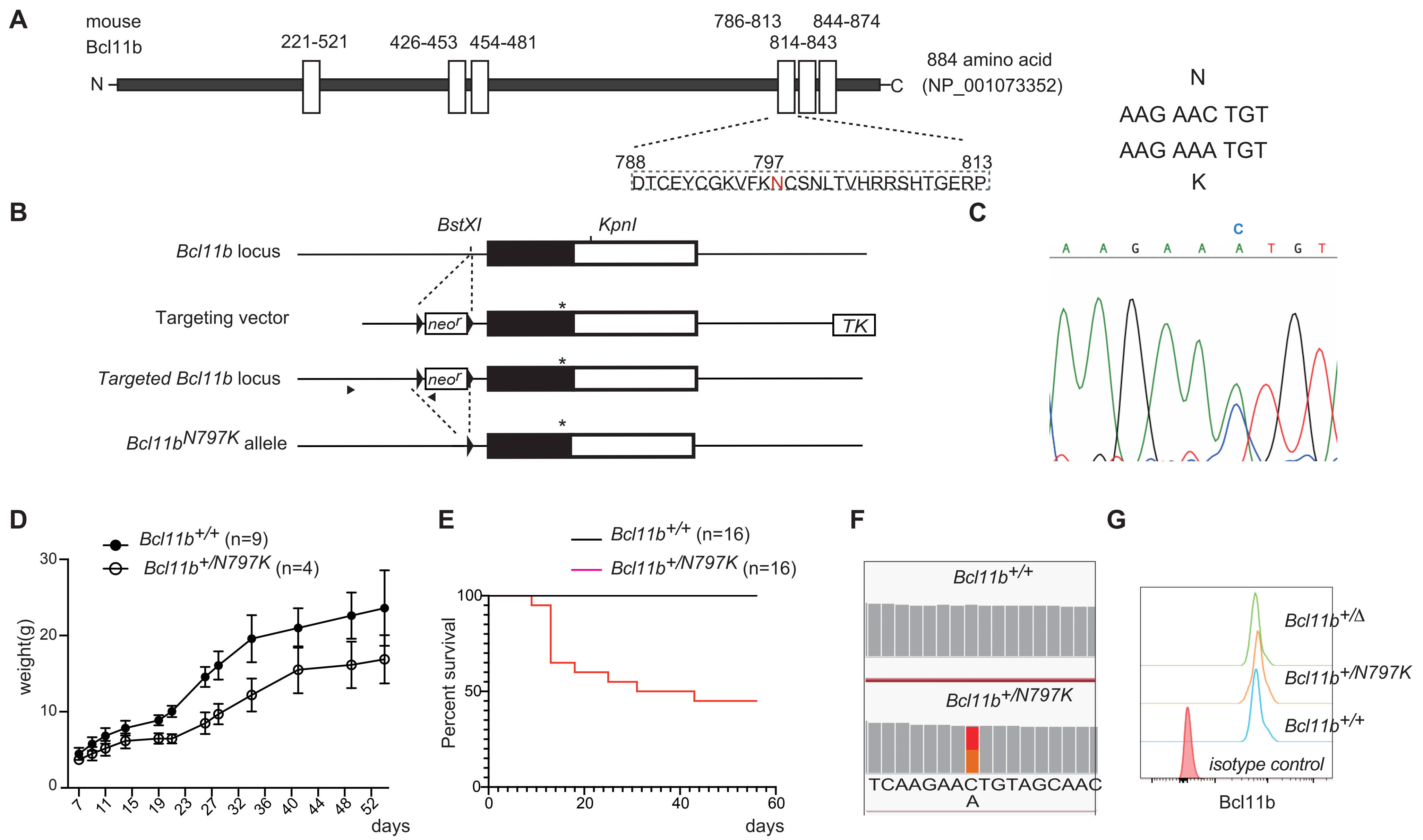

Supplemental Figure S1. Generation of *Bcl11b*^{N797K} mouse strain.
A.The scheme of murine Bcl11b protein (NP_001073352) is shown. Open boxes indicate the positions of the six C2H2-type zinc finger domains. The amino acid sequence nearby the N797 (red) residue replaced with K is shown. The designed DNA sequences for the C to A replacement to generate the *Bcl11b*^{N797K} allele are shown on the right. **B**.The strategy of Bcl11b targeting is shown in this scheme. Asterisks and triangles indicate the position of the introduced N797K point mutation and the designed primers, respectively. *: the N797K mutation. *neor*: neomycin resistant gene, *TK*: thymidine kinase. **C**.Sanger histogram of the genome extracted from the *Bcl11b*$^{+/N797K}$ ES clone confirming the C-to-A replacement. **D**.Body weights of *Bcl11b*$^{+/+}$ (n = 9) and *Bcl11b*$^{+/797K}$ (n = 4) F1 mice at the indicated postnatal dates are shown. **E**.Survival curves of *Bcl11b*$^{+/+}$ (n = 16) and *Bcl11b*$^{+/N797K}$ (n = 16) F1 mice is shown. **F**.Ratio of the read counts from the RNA-seq of total thymocytes from *Bcl11b*$^{+/N797K}$ mice showing the equal expression of the Bcl11b and *Bcl11b*^{N797K} allele. **G**.Histograms showing Bcl11b protein expression in total thymocytes from a *Bcl11b*$^{+/+}$, *Bcl11b*$^{+/\Delta}$ and *Bcl11b*$^{+/N797K}$ mice. Isotype control antibody is shown as negative control.

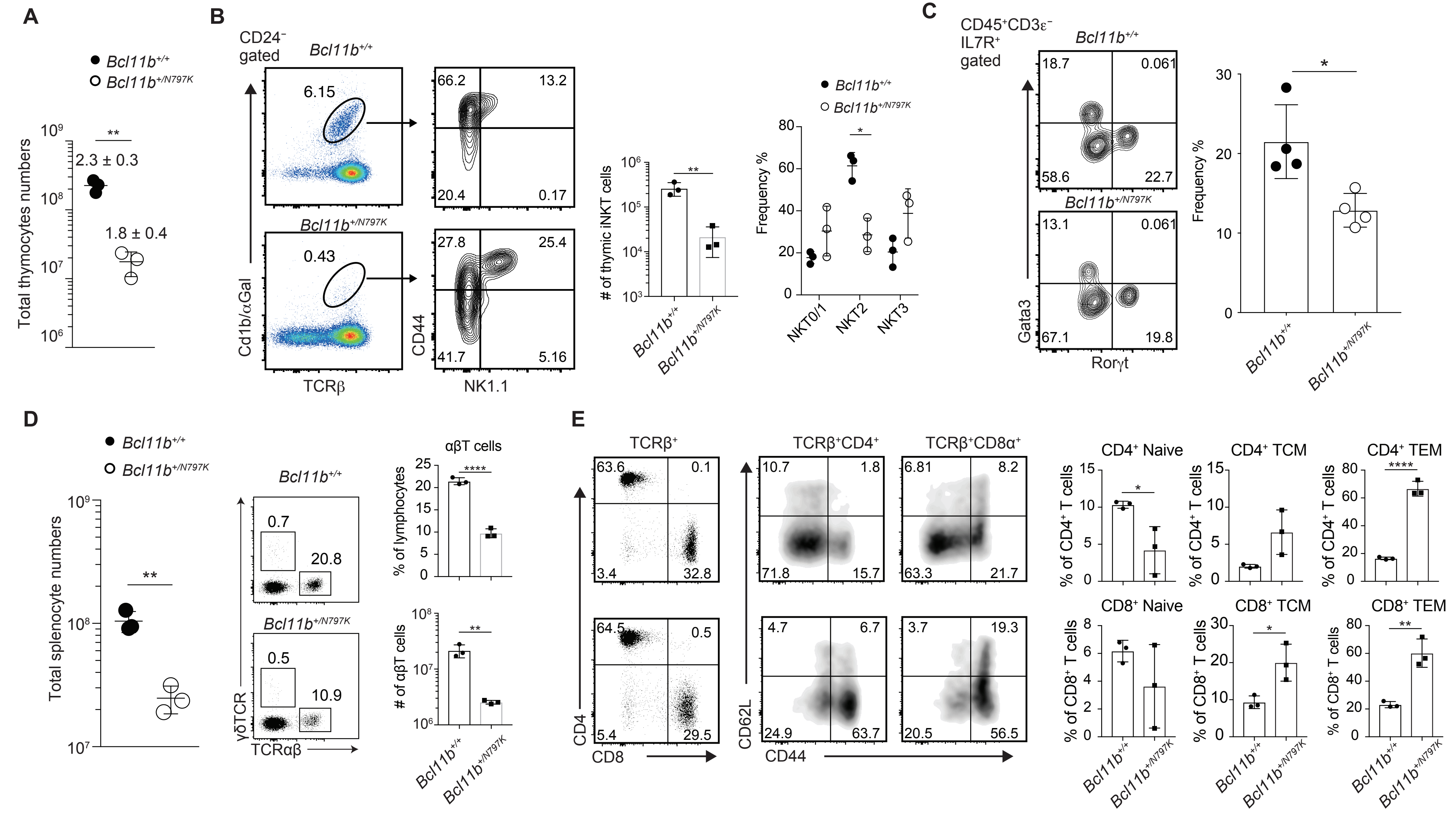

Supplemental Figure S2. Impaired T cell development in *Bcl11b*$^{+/N797K}$ mice.
A.Graph shows total thymocytes numbers of *Bcl11b*$^{+/+}$ and *Bcl11b*$^{+/N797K}$ mice. **B**.Representative pseudo-color plots showing the binding of CD1d-dimer loaded with αGalCer and anti-TCRβ Ab in CD24^{-} thymocytes. Contour plots showing CD44 and NK1.1 expressions in iNKT cells. Right graph depicting absolute number and the frequency of CD44^{-}NK1.1^{-} stage 0/1, CD44^{+}NK1.1^{-} stage 2 and CD44^{+}NK1.1^{+} stage 3 iNKT cells. **C**.Contour plots showing the expression of Gata3 and Rorγt of CD45^{+}CD3ε^{-}IL7R^{+} cells in the lamina propria of the small intestine of *Bcl11b*$^{+/+}$ and *Bcl11b*$^{+/N797K}$ mice. ILC2 cells were defined as a Gata3^{+} Rorγt^{-} cells. Graph showing the frequency of gut ILC2 cells. **D**.Left graph showing the total number of splenocytes of *Bcl11b*$^{+/+}$ and *Bcl11b*$^{+/N797K}$ mice. Dot plots showing γδTCR and TCRβ expression in total splenocyte and right graph showing the frequency and the numbers of splen αβT cells. **E**.Dot plots showing CD4 and CD8 expression in TCRβ^{+} splenocytes. Zebra plots showing CD44 and CD62L expression in TCRβ^{+}CD4^{+} and TCRβ^{+}CD8^{+} splenocytes. The graphs on the right showing the frequency of naïve (CD62L^{+}CD44^{-}), central memory (CM) (CD62L^{+}CD44^{+}), and effector memory (EM) (CD62L^{+}CD44^{-}) cell subset in splenic CD4^{+} and CD8^{+} T cells. All data in the graphs are presented as the mean ± S.D.; one-way ANOVA with Tukey' s post-hoc test; *: $p < 0.05$, **: $p < 0.005$, ***: $p < 0.0005$.

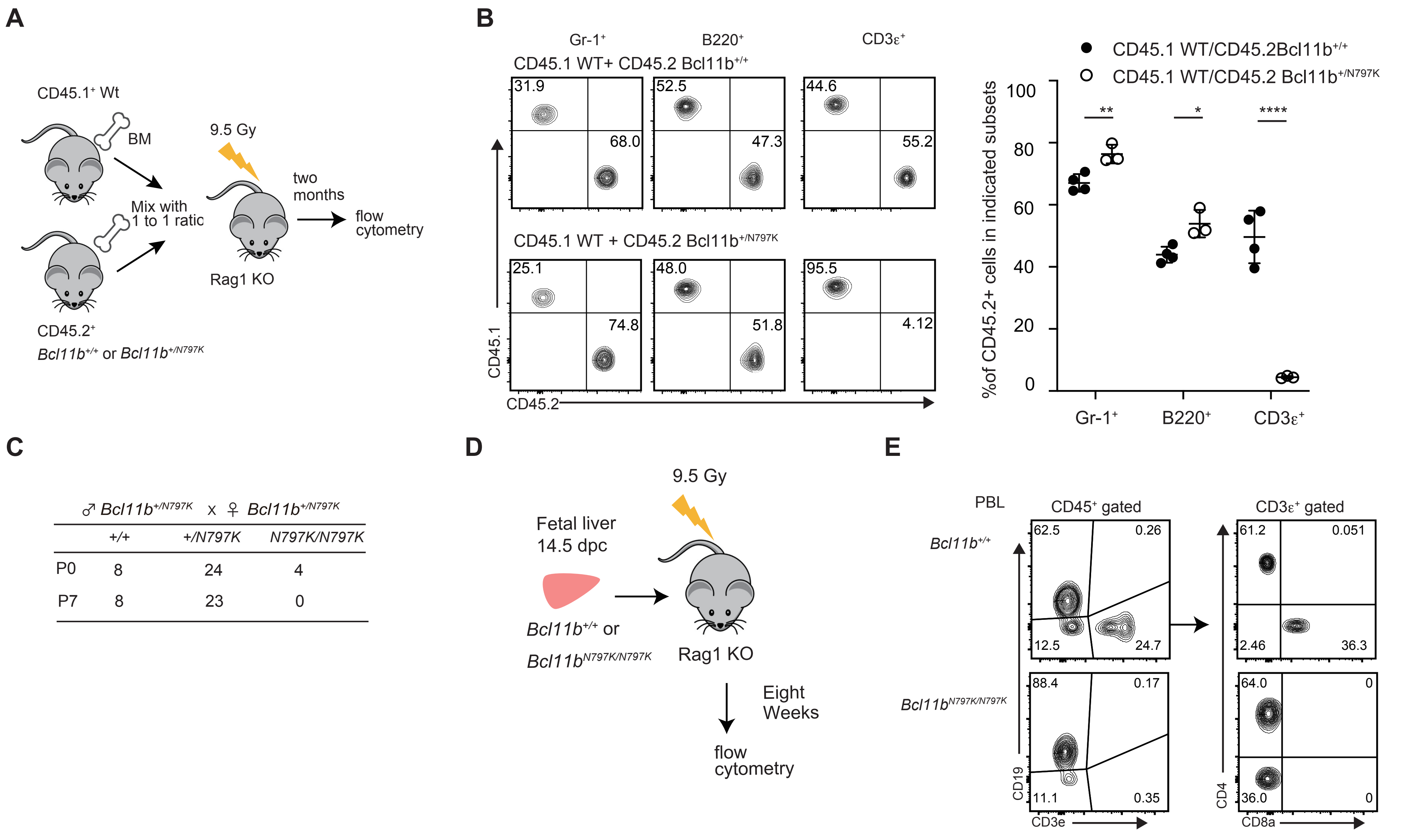

Supplemental Figure S3. T cell reconsitution from bone marrow and fetal liver.
A.A scheme showing the experimental flow for mixed bone marrow chimera using congenic CD45.1 and CD45.2 markers and lethally irradiated Rag1-defcient recipients. **B**.Contour plots showing CD45.1 and CD45.2 expressions in Gr-1^{+}, B220^{+} and CD3ε^{+} cells in the peripheral blood of recipient mice transplanted with CD45.1+ Bcl11b+/+ bone marrow cells and either CD45.2^{+} *Bcl11b*$^{+/+}$ or *Bcl11b*$^{+/N797K}$ bone marrow cells. Graph summarizing the frequency of CD45.2^{+} cells in the indicated cell subsets. Data are shown as the mean ± S.D. **C**.Table showing the number of live *Bcl11b*$^{+/+}$, *Bcl11b*$^{+/N797K}$ and *Bcl11b*$^{N797K/N797K}$ neonates at P0 and P7. **D**.A scheme showing experimental flow for reconstitution of hematopoiesis from fetal liver (FL) cells of 14.5-dpc *Bcl11b*$^{+/+}$ or *Bcl11b*$^{N797K/N797K}$ embryos.
E.Contour plots showing CD19 and CD3ε expression in CD45^{+} peripheral blood cells (PBL) and CD4 and CD8 expression in CD3ε^{+}CD45^{+} PBLs from sub-lethally irradiated Rag1-defcient mice transplanted with *Bcl11b*$^{+/+}$ or *Bcl11b*$^{N797K/N797K}$ FL cells.

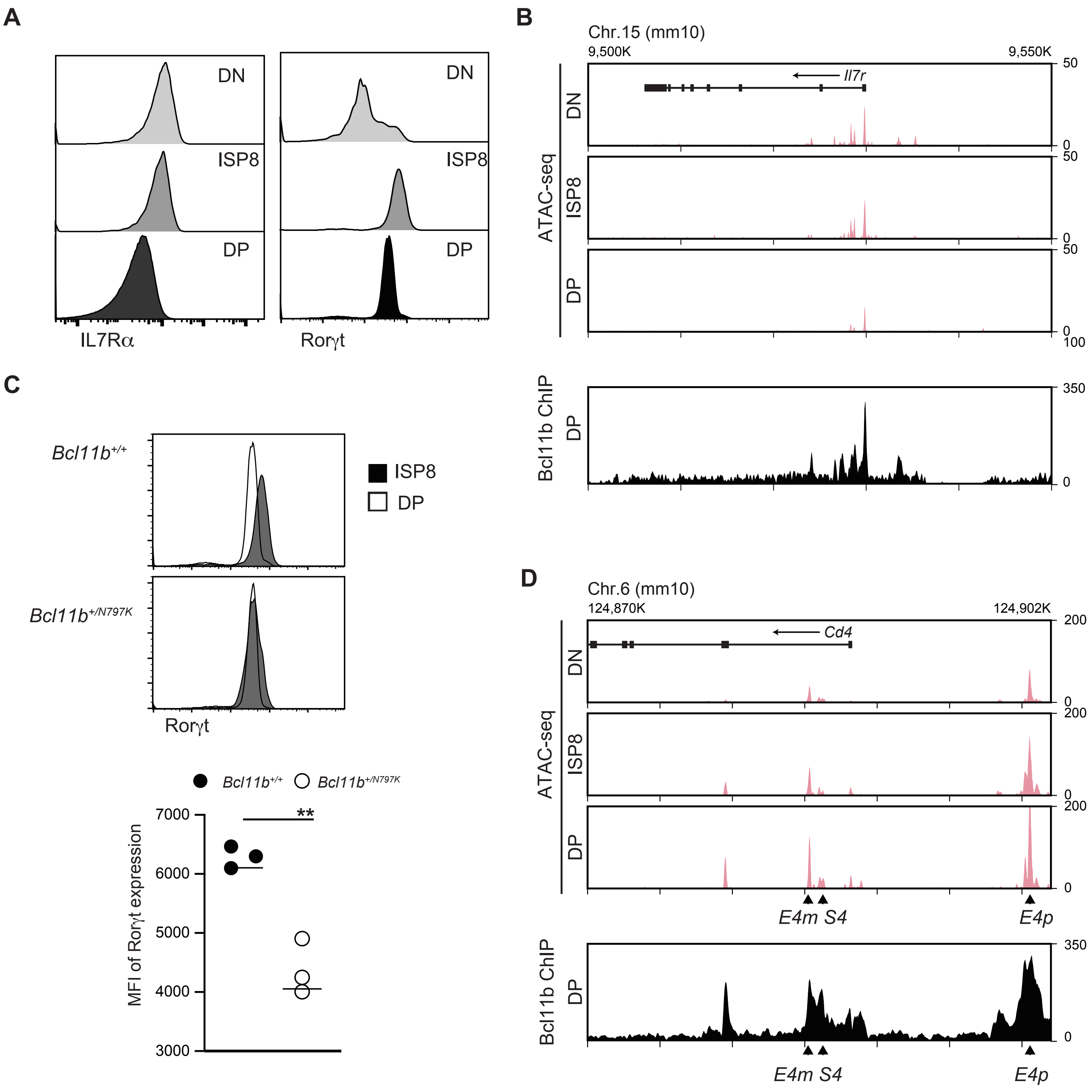

Supplemental Figure S4. impaired *Cd4* gene activation by at the DN to DP transition by *Bcl11b*^{N797K} mutation. **A**.Representative histogram showing IL-7Rα (left) and Rorγt (right) expression in CD4^{-}CD8^{-} DN, immature CD8 SP (ISP8) and CD4^{+}CD8^{+} DP thymocytes from wild type mice. **B**.ATAC-seq tracks at the *Il7r* locus in CD4^{-}CD8^{-} DN, ISP8 and CD4^{+}CD8^{+} DP thymocytes, and Bcl11b ChIP-seq track in CD4^{+}CD8^{+} DP thymocytes. **C**.Histograms showing Rorγt expression in ISP8 and CD4^{+}CD8^{+} DP thymocytes of *Bcl11b*$^{+/+}$ or *Bcl11b*$^{+/N797K}$ mice. Lower graph showing a mean fluorescent intensity of Rorγt expression in ISP8 thymocytes of *Bcl11b*$^{+/+}$ and *Bcl11b*$^{+/N797K}$ mice. **D**. ATAC-seq tracks at the *Cd4* locus in CD4^{-}CD8^{-} DN, ISP8 and CD4^{+}CD8^{+} DP thymocytes, and Bcl11b ChIP-seq track in CD4^{+}CD8^{+} DP thymocytes. The positions of the *E4p*, *E4m* and *S4* are indicated by the arrowhead. Bcl11b ChIP-seq data was from GSE90949. Unpaired t-tests were performed. ** $p < 0.005$
